# Codeine for Morphine Habit

**Published:** 1889-10

**Authors:** 


Codeine for Morphine Habit.—Dr. Constantine Schmidt, of Wiesbaden, publishes an article in *Notes on New Remedies* (Lehn & Fink) for September, 1889, entitled The Cure of the Morphine Habit, in which he advocates the employment of codeine therefor. After reducing the morphine to a very small dose by gradual withdrawal, he substitutes codeine, employing only as much as seems necessary to relieve the symptoms consequent upon the withdrawal. The following words close the article:

As the latter grow weaker, and gradually vanish, the codeine is reduced proportionately until the last traces of the symptoms of abstinence, as well as the excitable nervous debility, disappear. Exceptionally there is an increase in weight, and in bodily and mental health. For these reasons, I feel justified in regarding the patients discharged as *cured* after this treatment, principally for the reason that for future occasions, instead of morphine, they have codeine as a remedy at their disposal, which is a certain safeguard against relapse. That the after-treatment must be directed towards the often long-lingering nervous weakness in all its various phases, and toward the antecedent diseases wherever such exist, is apparent, and needs no further comment. At all events, we must now recognize and intimately acquaint ourselves once for all with the idea, that for every habit there is but one cure, that the latter consists in conversion of the perceptibly growing weakness into gradually increasing strength, and that the successful treatment of the morphine patient, if its aim should *not* be a mere *withdrawal* but a real *cure*, must extend over several months, and can only be accomplished with *certainty* in an institution *specially* equipped, and under the guidance of a competent professional.

The author states that the codeine used by him is specially prepared by a peculiar process. Steps were at once taken by Messrs. Lehn & Fink to procure the special manufacture, and this variety of codeine can now be obtained in this country.

